# Using the VERA-2R, professional and organisational aspects

**DOI:** 10.3389/fpsyt.2023.1165279

**Published:** 2023-07-20

**Authors:** Nils Duits, Carina Overdulve, Maaike Kempes

**Affiliations:** Netherlands Institute of Forensic Psychiatry and Psychology (NIFP), Utrecht, Netherlands

**Keywords:** violent extremism risk assessment, VERA-2R, professional use, organisational aspects, judicial organization, risk management, risk transfer, training

## Abstract

**Introduction:**

Violent extremism risk assessments of individuals suspected or convicted of terrorism are relevant for legal decisions, in prison and probation settings, and in inter-professional risk collaboration. These risk assessment reports by professionals should be applicable to and usable for the different judicial contexts. Informal and formal clinical practice evaluations, in the form of practitioners feedback and standardised evaluation of professional violent extremism risk reports are needed to gain insight in the use and quality of violent extremism risk assessments.

**Methods:**

In this study we examined how forensic professionals from three different countries (Sweden, Belgium and the Netherlands) use the VERA-2R in different judicial contexts. We also investigated which organizational aspects are important for the use of the VERA-2R. We focused on the perspective of the forensic professionals and their judicial organisations. We did a standardised survey among 86 VERA-2R trained professionals and a standardised interview with 20 executives and managers of organizations working with the VERA-2R.

**Results:**

This study showed that professionals find the VERA-2R useful for structuring information and speaking a common risk language. However, using the VERA-2R comes with a variety of challenges, both on the professional and organisational level. VERA-2R trained professionals had few opportunities to use the instrument and when they did, they were not always offered regular supervision, intervision and booster training. Also, organisational issues in collaboration between judicial partner organisations and the lack of risk transfer information to professionals came to light.

**Discussion:**

More research on the topic of risk transfer is needed. Policy implications are advised, for example the development of booster trainings, more organizational support, regulations on re-assessments, providing expertise and knowledge to indirect stakeholders and clear writing guidelines.

## Introduction

Since the terrorist attacks in New York, Paris, London, Brussels, and many other cities, there has been an increased focus on countering violent extremism and terrorism. Countering terrorism is a global security challenge (1). Although many definitions and types of terrorism exist, one can define terrorism in a more general way as ideologically inspired (preparations for) the perpetration of acts of violence against human life or of acts society-disrupting damage, with the aim of creating a climate of serious fear among (part of) the general population, bring about social change and/or influence political decision-making (2-4). Therefore, being a member of or participation in a terrorist organization, threatening with terrorist attacks, recruiting, and financing terrorism are also considered to be terrorist offenses. Violent extremism can be described as the beliefs and actions of individuals who support or use violence to achieve ideological, religious or political goals (5).

Individuals who are imprisoned for a violent extremist or terrorist offence, as well as prisoners who are radicalised in prison, pose a serious security threat, both during their imprisonment and after their release from prison (6). The average prison sentence for terrorist offences in the reported proceedings in European Member States in 2021 was 6 years (6). Tackling potentially violent, extremist lone actors and safe reintegration measures for persons who have been convicted of terrorist offences, both during and after detention will require special attention. This is all the more important because individuals who are convicted of terrorist offences will regularly be released from detention in the years ahead (2).

This raises the question whether and how judicial professionals in prison and probation settings evaluate, report and supervise the risks of terrorist (re)offending and whether or how they communicate their risk assessments with and to other professionals. This requires evidence-based professionalism in violent extremism and terrorism risk assessment and risk management.

To assess the risks on violent extremism, the Violent Extremism Risk Assessment tool, the VERA, was developed by Elaine Pressman. It was identified as relevant for the terrorist population in an Australian high-risk correctional terrorism unit (7, 8). After feedback of professionals, and using scientific evidence, the risk assessment tool was revised and updated several times, resulting in the VERA-2R (9, 10). The VERA-2R offers evidence-based professionalism with indicators based on empirical and expert knowledge in radicalization, violent extremism and terrorism. These VERA-2R indicators turned out to be applicable to different terrorist offenses (10, 11). The VERA-2R tool can be used to assess the risk status and risk management at different stages within the criminal process when a person is accused, arrested or convicted of a violent extremist or terrorist offence, imprisoned or released from detention. Repeated risk assessments should also be made in the event of a change in the judicial situation and are certainly necessary when convicted persons return to society or during supervision by the probation service to re-examine the risk status over the course of time and eventually adapt the risk management (9, 10).

The VERA-2R is a Structured Professional Judgement (SPJ) instrument. SPJ does not rely on statistical assumptions and the application of group-based estimates to individuals. Rather, it relies on the ability of the evaluator to develop a meaningful appreciation for the risk propensity of the individual (9, 12–14). The SPJ method combines evidence-based knowledge about risk analysis and the principle of structured professional judgement. The final professional judgement is not determined by enumeration of the relevant risk factors, which means that the presence of more risk factors does not necessarily result in a higher overall risk (15). Following the SPJ methodology, the VERA-2R acknowledges that the weighting of the indicators should not be defined beforehand, because the relevance of the indicators may vary with the context of the individual (9). Therefore, SPJ is used to integrate, combine and weigh all relevant information related to the risk indicators. The value of using SPJ is that it provides a clear substantiation of how and why a risk assessor came to a particular decision based on the outcome of the risk indicators. This is important for those for whom the risk analysis is intended. A good substantiation also helps protecting the evaluator against unfair or unsubstantiated criticism later, should an individual reoffend. Engaging in SPJ and consensus meetings could contribute to the development of a shared perspective on risk management strategies, both within and across organizations. This could eventually facilitate the communication of risk assessment outcomes between organizations, leading to clarity of the risk assessment’s purpose (14).

The VERA-2R is widely used by trained professionals in and outside Europe to assist with decision-making in different steps of the criminal justice process. In recent years, the number of VERA-2R trained professionals in Europe has increased considerably to more than 1,000 professionals for different judicial purposes.^1^ To illustrate how the VERA-2R is used within different judicial contexts, the forensic practice of the Netherlands, Sweden and Belgium will be highlighted. Since 2016, using the VERA-2R is mandatory in the Netherlands and also in post-trial advice for the court in Belgium. In the Netherlands, the VERA-2R has to be used in a pretrial context, in forensic mental health advice reports for the court and in probation advice reports considering suspects of terrorist offenses (16). Subsequently, the VERA-2R must be used in the Netherlands in prison to compose risk profiles of persons suspected of terrorism for the purpose of ‘prison differentiation’ for the three Dutch terrorist prison wards, or in case of suspected radicalisation in prison. In the Netherlands the VERA-2R risk assessment is being shared more and more between judicial professionals (2). In Belgium, the VERA-2R has to be used in the judicial context and in the post-trial context, to advice on risk management in case of an offender’s (conditional) release. Since 2021, the VERA-2R has to be re-evaluated by the Flemish probation office, during parole supervision. The Belgian probation offices are defederalized, which means that every region (Flanders, Brussels and Wallonia) has their own regional jurisdiction. At this moment, only the Flemish Probation Office uses the VERA-2R. In 2018, The Swedish Prison and Probation Board was trained in the VERA-2R. Risk assessment is mandatory, given that a convicted terrorist offender is sentenced for at least 4 years of imprisonment. These offenders are assessed in a specialized unit, where psychological risk assessments are a mandatory part of the detention planning. Otherwise, the VERA-2R is used in advice reports that consider the probation planning of convicted terrorist offenders or potential radicalized inmates.

An intensive theoretical and case-based training using a comprehensive manual (which exists in four languages) with all coding principles, is obligatory to use the VERA-2R as a SPJ tool to obtain analytic skills, to obtain confidence in violent extremism risk assessment and risk management, how to write risk reports, and how to evade common pitfalls^2^ (9). Supervision, intervision and feedback by more experienced professionals can improve violent extremism risk assessments of VERA-2R trained professionals (9, 14).

Although risk assessment tools and their psychometric properties are widely studied, more focus is needed on the professional perspective and competence of working with these tools (17, 18). In general, professionals need a mandate of their government or professional agency to conduct risk assessments, and they need to follow and complete a recognized training program. They should also have theoretical knowledge, and expertise in forensic assessments, and experience with the evidence based practice of the SPJ method of risk assessment and risk management. Furthermore, they must also understand the advantages and caveats of the specific risk assessment tool, in terms of its application, and be able to explain these limitations and advantages (9, 18). There are other clinical challenges as well for professionals. Experience may differ between the different disciplines involved, as well in relation to the legal context in which the risk assessment takes place (17). It may be difficult to carry out proper forensic investigations into terrorists and violent extremists in a judicial setting, partly depending on the judicial facilities, lacking information or time-consuming assessments (19). Also, reintegration of terrorist and violent extremist offenders needs cooperation of different judicial partners, and is not yet based on evidence-based practice (20, 21).

Therefore, it seems that only evaluating the outcome of the use of risk assessments in terms of risk reports, might not fully reflect their user validity. To ensure the user validity of a risk assessment tool, such as the VERA-2R, research emphasizes the importance of practice evaluations. For example, asking practitioner’s feedback and evaluating the way in which organisations support their professionals and engage in good practice standards (22, 23). Practice evaluations are in particular important for violent extremism risk assessments, as decisions of significant impact are based upon the outcome of these assessments (9, 16). In sum, a risk assessment tool may be a valid and reliable instrument, but without knowing how the tool is actually used in professional practice, it remains unclear whether the instrument is being used as intended (18, 21, 24). This emphasizes the need to study both the professional and organisational perspective on the use of the VERA-2R in different settings.

In this paper we examine how the VERA-2R is used in different judicial settings from a professional and organisational perspective. We investigate (1) how professionals use the VERA-2R in their professional practice and (2) which organizational aspects are important for applying the VERA-2R in professional practice. Our focus on the professional and organizational perspective relates to structural and processual aspects that are found to be important according to a forensic evaluation matrix model of quality with quality indicators in four perspectives: person or evaluee, professional, organisation, government, and three aspects: structure, process, outcome (25, 26). The structural professional perspective relates to quality indicators as professional’s expertise and experience, and the processual professional aspect relates to quality indicators as the professional’s efficacy, diligence, use of the available forensic instruments, communication, and collaboration. Quality indicators of the structural organisational aspects relate to sufficient resources, skilled professionals, good services, information and ICT. Quality indicators of the processual organisational aspects relate to avoiding of errors or delays, coordination between stakeholders, following protocols and rules, and innovation and development of instruments.

After clarification of the user validity of the VERA-2R, improvement and implementation of evidence based practice principles will be possible and necessary in different professional settings. In general, it is known that ‘risk assessment matters but only when implemented well’ (27) and that implementation of risk assessment tools used by professionals is complicated, especially when these tools are applied in different settings and serve different purposes (26, 28), also combining it with neurobiological risk indicators (29) Implementation outcomes relate for example to the acceptability, adoption, appropriateness, cost, feasibility, fidelity and penetration and sustainability of and to the instrument and all practical issues related to risk management and risk communication (18, 21, 22, 24, 27–31).

## Materials and methods

### Use of the VERA-2R, questionnaire, and interviews

#### Participants and judicial organisations

As explained above, VERA-2R training and use of the instrument is mandatory for forensic professionals working in the different judicial organizations in the Netherlands, Belgium and Sweden.

In the Netherlands, the VERA-2R has to be used by forensic psychiatrists and forensic psychologists of the Netherlands Institute of Forensic Psychology and Psychiatry (NIFP) in pre-trial forensic reports for court of persons suspected of terrorist activities. Social workers of the Dutch Probation Service have to use the VERA-2R for risk assessments of persons being placed in detention. Psychologists within the Dutch terrorist prison wings of the Dutch Custodial Institutions (DJI) have to use the VERA-2R for risk assessments related to prison differentiation in the different terrorist wards (leaders and followers and/or individuals who are negatively influenced). Psychologists and social workers of the Belgian Federal Public Service (FOD) have to use the VERA-2R in detention and for advice on the terms and conditions for parole release for the criminal execution court. The Flemish Probation Board uses the VERA-2R for risk assessment in probation planning. The Walloon probation service does not use the VERA-2R. Psychologists of the Swedish Prison and Probation Board use the VERA-2R for risk assessment of prisoners in detention, for prisoners who are eligible for conditional release, and for probation planning. The Dutch Probation Board intends to invest in repeated risk assessment during parole and probation supervision.

Professionals from the Netherlands, Belgium and Sweden who were trained by the NIFP in the VERA-2R between 2015 and 2021 were approached by e-mail to participate in the study. We sent the survey to 281 VERA-2R trained professionals; 139 of them were based in the Netherlands, 12 in Sweden and 130 in Belgium. Eventually, 86 VERA-2R trained professionals (31%) completed the questionnaire; 30 Dutch (22%), 34 Belgian (Flemish speaking), 15 Belgian (French speaking; 38%), and 7 Swedish (58%).

Additionally, 20 interviews were conducted with both executives and managers of organizations from the three different countries and judicial settings mentioned above working with the VERA-2R or practitioners involved with the implementation and supervision of the VERA-2R. Interviewees were based in the three different countries and in different judicial settings. The Netherlands with departments of the Dutch Custodial Institutions (DJI), the Netherlands Institute of Forensic Psychology and Psychiatry (NIFP), and the Dutch Probation Board. Belgium with the Federal Public Service (FOD) and the Flemish Probation (Justitiehuizen). Sweden with the Swedish Prison and Probation Board. Of these executives 13 were based in the Netherlands, 5 in Belgium and 2 in Sweden (Table 1).

**Table 1:** Level of management interviewees per country.

	Higher management	Middle management	Operational level
Netherlands	4	5	4
Belgium	2	0	2
Sweden	0	1	1
Total	6	6	7

### Materials

The questionnaire was designed in Adobe Pro and distributed to trained professionals in either English or French. The questionnaire consisted out of 36 questions with a mixed format; 10 statements, 20 multiple choice and 6 open questions (see Appendix A). The statements could be answered on a scale of: Strongly agree, agree, neutral, disagree and strongly disagree.

The questions were clustered in eight themes: 1. General questions such as year of VERA-2R certification, country of employment and risk assessment context, 2. Experience with risk assessment tools and Structured Professional Judgement, 3. VERA-2R assessment experience such as the frequency of VERA-2R use and the experienced usefulness, 4. VERA-2R training such as knowing how to use the tool in practice after training, availability of intervision and supervision, 5. VERA-2R reporting with the use of a writing format,6. VERA-2R personal user experience with opinions on time and opportunity to use the VERA-2R, 7. VERA-2R within your organization with the availability of consensus meetings, 8. Risk communication whether or not the VERA-2R is used to communicate risks to third parties.

### Procedure

Email invitations for the questionnaire were developed in Dutch, English and French. They included information about the study, and a document about how privacy is guaranteed. Also, an informed consent sheet was included, to formally gain the permission of the respondents that their answers are being used for this research project. Project participants and executives of the NIFP, the Dutch Probation Board, the Belgian Federal Public Service (FOD), the Flemish Probation Board and the Swedish Prison and Probation Board were approached by e-mail. They were informed about the questionnaire and encouraged to distribute the survey among their VERA-2R trained personnel. Subsequently, managers, executives and practitioners that were actively involved with the implementation of the VERA-2R were asked if they could participate in an interview to gain more insight on using the VERA-2R within their working context. Interviews were scheduled and conducted via Zoom and each took 20 to 30 min.

### Statistical analysis

Interviews have been transcribed verbatim. By using the coding program Atlas.ti, themes were used to analyse the interviews (see Table 2). Interview content that matched with a theme, was added to that theme with a code.

**Table 2:** Themes interview coding scheme.

Implementation
Characteristics VERA-2R assessor
Organizational Context VERA-2R use
Experienced Usefulness VERA-2R
Intervision VERA-2R use
User Barriers VERA-2R
Organizational Perspectives on Increasing User Value
Transfer of risk information
Recipient VERA-2R report

## Results professional use of the VERA-2R

### Demographic characteristics

Table 3 shows the distribution of the years in which the professionals completed the VERA-2R training.

**Table 3:** Year of VERA-2R training.

Year	Frequency
2015	11 (12.80%)
2016	15 (17.40%)
2017	9 (10.50%)
2018	24 (27.90%)
2019	10 (11.60%)
2020	8 (9.30%)
2021	9 (10.50%)
Total	86 (100%)

Professionals that participated in VERA-2R trainings are mostly psychologists, social workers or psychiatrists. In the Netherlands, other disciplines are also trained (See Table 4).

**Table 4:** Expertise VERA-2R professionals per country.

	Psychologist	Social worker	Psychiatrist	Prison official	Police officer	Intelligence	Law/Other	Total
Belgium Flanders	16	13	1			1		31
Belgium Wallonia	8	5	1					14
Sweden	6						1	7
Netherlands	12	7	3	2	2	2	2	30
Total	42	25	4	2	2	3	3	82

### Frequency of VERA-2R assessment

Table 5 displays the frequency of VERA-2R use in Belgium, Sweden and the Netherlands. Overall, 29 professionals (33.77%) have not worked with the VERA-2R in the last 2 years, 13 (15.10%) once and 26 (30.20%) used the VERA-2R two to four times in the last 2 years.

**Table 5:** Frequency VERA-2R use last 2 years per country.

Frequency	Never	Once	2–4	5–10	10–15	>15
Belgium Flanders	13	6	4	7	2	2
Belgium Wallonia	4	5	4	2		
Sweden	3		4			
Netherlands	9	2	14	4	-	1
Total	29	13	26	13	2	3

The reason professionals give for using the VERA-2R once or never in the last 2 years is mostly that they got no cases assigned, which was mentioned by 24 professionals. One of them stated: ‘There are few terrorism cases at the prison where I work and more staff has been trained in recent years. So the number of terrorism cases per person is low’. Other reasons of limited use of the VERA-2R are job rotations after VERA-2R training (32), no direct contact with clients due to involvement in policy and case supervision (32), recent completion of VERA-2R training (19), using another risk assessment tool for extremism (30) or due to insufficient information on a case (30).

### VERA-2R guidance and continuous learning

Professionals were asked whether or not they received supervision and intervision after they were trained. Table 6 shows that participants report that they did mostly not receive supervision. Intervision is only available for half of the professionals, taking together all countries. In each country, the majority of professionals indicate that they did not benefit from supervision. Furthermore, half of the professionals indicate that they did benefit from intervision. In Sweden and the French speaking part of Belgium a slight majority of the participants indicated that they did benefit from intervision.

**Table 6:** Availability of VERA-2R supervision and intervision.

Country	Supervision		Intervision		Total
	No	Yes	No	Yes	
Belgium - Flemish speaking	22	12	20	13	34
Belgium - French speaking	11	4	6	9	15
Sweden	5	2	2	5	7
The Netherlands	24	6	15	15	30
Total	62	24	43	43	86

Participants who report that they did receive supervision and intervision were asked to specify its form and frequency. Participants mention that they received supervision in the form of assessing the VERA-2R under supervision of other trained personnel. Also, they report that supervision includes receiving feedback on their risk assessment and the lay out of their report. The form of intervision mostly includes case discussion with colleagues.

The frequency of intervision differs between contexts, e.g.; three to four times during the first pilot year (Sweden), twice or three times a year (NIFP), monthly peer consultations (Belgian Federal Public Service; the Flemish Probation), extremist case discussions every 2 weeks (Dutch Probation Board).

Professionals reported continuous learning aspects related to the VERA-2R (see Table 7). In the survey, a list of learning aspects was provided with the possibility of adding personal suggestions. On every aspect, more than half of the professionals report the need of continuous learning possibilities, except for the themes SPJ-method, psychopathology and lone actors. No substantial differences are detected when controlling for country or working context. Other themes that professionals mentioned are: continuous learning on Islamism, how to deal with high risk VERA-2R scores and how to communicate risk. Specifically for the Dutch pre-trial context a participant suggests continuous learning on how to discriminate between what is mostly driven by antisociality or ideology and to specify criteria for when there are indications of mental health issues that might influence the tendency towards violent extremism.

**Table 7:** Continuous VERA-2R learning aspects needed by professionals (*N* = 86).

Theme	Needed	
	Yes	No
Practice	67 (77.90%)	19 (22.10%)
Risk scenarios	57 (66.30%)	29 (33.70%)
Literature	53 (61.60%)	33 (38.84%)
Risk reporting	52 (60.50%)	34 (39.50%)
Risk management	52 (60.50%)	34 (39.50%)
Right-wing extremism	47 (54.70%)	39 (45.30%)
Risk communication	43 (50.00%)	43 (50.00%)
Left wing extremism	43 (50.00%)	43 (50.00%)
Lone actors	40 (46.50%)	46 (53.50%)
SPJ-method	33 (38.40%)	53 (61.60%)
Psychopathology	29 (33.70%)	57 (66.30%)

### VERA-2R user experience

33 of the 57 professionals that reported to have worked with the VERA-2R once or more in the last 2 years elaborated on their user experience. Regardless of the country and working context, professionals using the VERA-2R report that being able to do the assessment in a structured way is very helpful, as stated: ‘I find the structure helpful, with items that you evaluate separately’. Professionals also mention that the VERA-2R manual and the example questions give clear guidance and definitions, it allows them to speak a common language, which helps in risk communication. Despite these positive aspects, professionals also report some difficulties with the use of the VERA-2R in practice.

Overall, professionals find it challenging to form conclusions and to differentiate between low, medium or high scores on VERA-2R indicators. Some professionals reported that differences exist between colleagues on weighting the indicators of the VERA-2R, as one states: “It can be tricky to judge which items weigh more, and it would be helpful with clearer guidelines for setting the risk level.”

Some professionals reported the challenge of assessing the VERA-2R based on few case information, for example with potential radicalized individuals, that do not express or disclose anything or with individuals that do not have a prior criminal history. One of the respondents stated: ‘The VERA-2R requires much information in order for it to work; most times, information is not available. That does not mean, however, that the method has no benefits, on the contrary, I find the SPJ framework very useful’. Some professionals mentioned that they sometimes doubt what information from the individual’s history can be used for coding some VERA-2R indicators. Other challenges were reported within specific working contexts. A Dutch professional involved in the ‘prison differentiation’ context reported being unsure how long a risk assessment would be valid, before a re-assessment is needed. Furthermore, in the Dutch probation context, professionals reported to experience difficulties with the application of the VERA-2R scenarios and risk management on an individual that is suspected or convicted for something else than jihadism, especially now that cases of right wing extremism are increasing. Another remark was: “It would be helpful to receive refresher/training on the use and scoring of the VERA-2R on a regular basis (e.g., annually)’.

Table 8 displays the level of agreement on statements about the VERA-2R training and its use. Overall, professionals agreed with the statements.

**Table 8:** Statements on professional’s satisfaction with the VERA-2R (*N* = 86).

Statement	Scale	Frequency
The VERA-2R SPJ method enables a good risk assessment and risk management of an individual	Strongly agree	7 (8.10%)
	Agree	63 (73.30%)
	Neutral	12 (14.00%)
	Disagree	4 (4.70%)
	Strongly disagree	0
The VERA-2R helps to structure the necessary information on violent extremism risk assessment and risk management of a subject	Strongly agree	18 (20.90%)
	Agree	60 (69.80%)
	Neutral	6 (7.00%)
	Disagree	1 (1.20%)
	Strongly disagree	0
	Missing	1 (1.20%)
The VERA-2R training was useful for me	Strongly agree	33 (38.40%)
	Agree	46 (53.50%)
	Neutral	7 (8.10%)
	Disagree	0
	Strongly disagree	0
After training I knew how to use the tool in practice	Strongly agree	9 (10.50%)
	Agree	67 (77.90%)
	Neutral	5 (5.80%)
	Disagree	5 (5.80%)
	Strongly disagree	0

## Results organizational perspectives on VERA-2R use

Interviews regarding the organisational perspectives of the VERA-2R were conducted with Dutch participants working for the NIFP (*N* = 4), the Dutch Probation Board (*N* = 3), Program Approach to Radicalization & Extremism (PARE; *N* = 2) and Prison Terrorist Units (*N* = 3). Belgian participants worked for Cell Extremism (*N* = 1), The Psychosocial Service (PSD) of the Federal Public Service (*N* = 3) and Flemish Probation Board (*N* = 1). Both Swedish participants were working for the Swedish Prison and Probation Board (*N* = 2).

### VERA-2R implementation

Most organisations have created a central service for cases that need to be evaluated with the VERA-2R, except for the Swedish and Probation Board. The Swedish Prison and Probation Board has a specialized prison unit, for psychological risk assessment for those with a sentence of at least 4 years. When these offenders are suspected to be radicalized or sentenced for a terrorist offense, then the VERA-2R is also assessed. Within other countries and departments these central services are used for assigning cases to VERA-2R trained personnel and providing feedback on their reports. The organisations overall use a fixed format for their VERA-2R reports. The NIFP does have a standard, but they are used differently, as stated by one of their supervisors ‘Some professionals report on all the VERA-2R factors very extensively and others keep it very concise’.

### Characteristics VERA-2R assessor

In the Netherlands the VERA-2R is used by amongst others psychologists, psychiatrists, and probation officers. These were the professions included in this study. In Belgium psychologists and psychiatrists assess the VERA-2R, with the assistance of prison social workers. Probation workers also assess the VERA-2R in Belgium. In Sweden, only psychologists within the prison setting use the VERA-2R. These professionals were all familiar with other risk assessment instruments, except for probation workers working for the Flemish Probation Board.

### Experienced usefulness VERA-2R

Interviewees mostly mention that the VERA-2R is experienced as a useful tool as it promotes a structured working method and that it creates a common risk language. However, the usefulness differs between contexts. For the probation context it is mentioned that the VERA-2R helps to advice on suitable terms and conditions for release, whereas for the Dutch terrorist prison wings the VERA-2R is considered useful to gain insight into the network of the detainees, specifically the dissemination of radical ideas related to violent extremism. Because of these differences in context one of the directors of the Dutch terrorist prison wings mentions: ‘I do think it helps that professionals within the prison use the VERA-2R themselves, instead of only depending on the VERA-2R reports of the probation service, as the probation service has a different role than the prison’. In the past, psychologists of two Dutch prison wings complete the initial risk profile of the Dutch Probation Board during detention based on information received and their own observations. However, currently not the psychologist but the case manager, uses the VERA-2R in case a detainee is transferred to another setting.

### Organizational support VERA-2R use

Not all organizations engage in specific support related to the VERA-2R. The NIFP and the Belgian Federal Public Service offers general supervision in terms of professional feedback on reports, but there is no intervision. However, the NIFP is now setting up intervision. Within the Dutch Probation Board and Swedish Prison and Probation Board the VERA-2R is part of case discussions, but not on a regular basis. The Flemish Probation Board does offer monthly intervision with case discussions, in which the application of the VERA-2R is specifically discussed.

Additional organisational support involves the availability of additional hours for a VERA-2R assessment, specifically within The Flemish Probation and the NIFP. Also, the Belgian Federal Public Service has assembled its own additional training for professionals that are trained in the VERA-2R by the NIFP. This training involves 8 days, in which themes as radicalization processes, polarization dynamics and extremist groups are highlighted. Professionals are introduced to the Belgian system and organizations involved with extremism, to get familiar with their working context and the information flux between organizations.

### User barriers

Overall, managers do report that the whole process of the VERA-2R assessment is considered time-consuming for their professionals. Managers of the Netherlands (NIFP and Dutch Probation Board) and the Swedish Prison and Probation Board mention that their professionals find it hard to apply the VERA-2R on cases involving right wing violent extremism. Also, managers do report there is a lack of common understanding on the definition of violent extremism. As the VERA-2R is only assessed when someone is suspected or convicted for an extremist offense, organizations working with the VERA-2R are dependent on incoming cases that have been classified as violent extremism related. It is also mentioned that the VERA-2R is not considered to be fully compatible with a purpose of an organizational context, for example in the Dutch terrorist prison wings: ‘Even though we would like the VERA-2R to be universal, it is not fully compatible with how we differentiate inmates’. The Dutch Probation Board delivers their risk analysis reports within four to 6 weeks after a suspect’s placement, but these reports are sometimes of limited use to the Terrorist prison wings. The report is often incomplete or perceived as uninformative for detention differentiation. However, it is also mentioned that in this early stage of an individual’s criminal process one deals with a limited amount of information than can be used for an informative risk analysis.

Another organizational barrier is the collaboration between judicial organizations in each country. For example, in Sweden the intelligence service orders the prison to do a VERA-2R assessment in case of suspected radicalized inmates, but the intelligence service is not allowed to share information that corroborates this suspicion. In Belgium, it is difficult to get hold of all the relevant judicial documents to assess the VERA-2R, because these documents have to be physically obtained at the clerk’s offices. In the Netherlands, the Probation Board, the NIFP and the Terrorist prison wings assess the VERA-2R during the same time frame, but these organizations do not always possess the same information and their conclusions on the VERA-2R sometimes differ.

A context specific barrier is the minimal presence of violent extremism cases in Sweden, which makes it hard for psychologists to maintain their expertise. Another context specific issue involves the Dutch Terrorist prison wings, where it is not yet common practice to update or reassess the VERA-2R during someone’s imprisonment. This means that the advice on possible terms and conditions during parole supervision after months or years is based on information at the beginning of someone’s sentence. After the interviews, a director of a Dutch Terrorist prison wing added that they agreed upon a yearly VERA-2R update for all detained extremist and terrorist individuals. However, this agreement is only made in that specific terrorist prison wing.

### Organizational perspective’s on increasing user value

To increase the user value of the VERA-2R, the need for more frequent theoretical and practical (booster) VERA-2R trainings is expressed within all contexts. Also, the need for a universal working method with respect to violent extremist cases and terms of VERA-2R use within judicial departments and institutions is mentioned.

To enhance the user value of the VERA-2R, participants suggested to invest in training for indirect involved organisations, who are at the receiving end of a VERA-2R analysis.

Specifically, in the Dutch prison and probation context the need for guidelines on when to do a reassessment of the VERA-2R is mentioned. A manager of one of the Dutch terrorist prison wings suggested that the risk assessment should be assessed by someone else than the involved psychologist that gives treatment within the prison, to overcome potential bias.

### Transfer of risk information

With respect to the transfer of risk information there are several differences between countries. In Belgium, the Federal Public Service share their risk reports with the Flemish Probation Board when individuals are about to be released from prison. Next to these risk reports, the Flemish Probation Board also receives the completed assessment form of the Custodial Services, with the coding on each VERA-2R domain and indicator. During parole supervision, the Flemish Probation Board re-evaluates these VERA-2R domains and indicators and compares them with the coding of the previous report. This comparison is described in their parole risk management reports. In the Netherlands, the prisons do not share their previous VERA-2R risk assessments and other risk related information with the Dutch Probation Board, mostly due to privacy issues. For example, the psychologists in prison are licensed mental health professionals that also use the VERA-2R. VERA-2R related information is therefore considered to be confidential, because the professionals appeal to their medical confidentiality agreement. Only in one (small) terrorist wing of the three Dutch terrorist prison wings the probation officers administer the VERA-2R themselves for prison differentiation. Only there, they can retrieve the risk information for parole issues when someone is getting (conditionally) released from prison. In the other wings, no risk information is transferred for parole issues upon (conditional) release. In Sweden, the Swedish Prison and Probation Board shares their risk reports when an individual is about to be released from prison, with the responsible probation officer. The probation officers, however, do not yet use the VERA-2R for repeated risk assessments during the supervision and monitoring trajectory.

### Recipient VERA-2R report

To evaluate organisational differences in the exchange of VERA-2R reports, interviewees were asked about the recipients of their reports. Within Belgium, VERA-2R information concerning prisoners is shared with Cell Extremism, part of the Belgian Prison System. Cell Extremism shares relevant information with external intelligence services. Also, risk reports that contain the outcomes of a VERA-2R analysis are shared with the Belgian court for the executions of sentences, in the context of parole decisions. The Swedish Prison and Probation Board shares their VERA-2R reports with their probation service. Within the Netherlands, the NIFP and Dutch Probation Board share their reports with the public prosecution office and Dutch court. The Dutch prisons share their VERA-2R related information on detainees with another prison in case of a transfer. Relevant VERA-2R information is not always shared with the Dutch Probation Board, in case of parole or release from prison.

## Conclusion

This study aimed to evaluate the current practice of the VERA-2R, by addressing the structural and processual aspects of the forensic professional and organisational perspectives (25, 26). We focused on judicial professionals and judicial organisations that work with the VERA-2R. We examined the user experience of the VERA-2R among trained professionals in Sweden, Belgium and the Netherlands and secondly, we examined how the VERA-2R is used in violent extremism risk reports across different judicial contexts within these countries.

In relation to the professional context we can conclude that a variety of trained professionals use the VERA-2R for different judicial purposes. The trained professionals within these three countries have not used the VERA-2R very often in the past 2 years. In fact, a third of all respondents did not use the VERA2R at all. This was mainly due to not getting assigned to violent extremism related cases. In addition, more than half of the professionals did not receive regular supervision and half of the professionals received regular intervision related to their VERA-2R use. Regarding user experience, professionals find the VERA-2R useful for structuring information and for speaking a common risk language. Some professionals reported some difficulties with the interpretation of the VERA-2R indicators and differentiation between them. These conclusions applied to every country and working context.

In relation to the organizational context we can conclude that the organisational barriers that interfere with the user validity of the VERA-2R were rather context specific. For example a low amount of violent extremism or terrorism related cases in Sweden, and no updated VERA-2R assessments in the Dutch prison and probation context. Regardless of the judicial context, difficulties in obtaining risk information from other organisational partner organisations was experienced as a critical organisational barrier. This non-transfer of violent extremism or terrorism risk information and risk reports was an important issue in the Dutch judicial situation. To obtain this information was either difficult and time-consuming for professionals in the beginning of the judicial process or the sharing of risk information was not considered possible due to presumed privacy issues. These organisational issues can pose an unknown risk of recidivism if convicted terrorists or violent extremists are (conditionally) released and have to be accompanied during their parole. Several practical recommendations were made. In particular, to offer training to personnel who are indirectly involved in the VERA-2R assessment process, for example, persons and organizations that receive and have to use VERA-2R risk reports. Managers also talked about the need for refresher training for VERA-2R trained professionals, both on the topic of violent extremism and terrorism and the use of the VERA-2R itself.

## Discussion

The majority of the conclusions related to the user validity of the VERA-2R apply to all three countries (Sweden, Belgium and the Netherlands) and all different judicial contexts addressed in this study.

With respect to the professional perspective, VERA-2R trained professionals generally reported having little VERA-2R user experience. This is mainly due to getting assigned too few violent extremism or terrorism related cases. This may be because there are relatively few terrorist cases, the VERA-2R is not used regularly enough, and/or because there are too many VERA-2R trained professionals related to the case load. From the organizational perspective of quality, it was reported that external parties, such as an intelligence service or a prosecution office, determines which extremist or terrorist cases need a VERA-2R assessment. It is likely that there is a gap between the existing extremist or terrorist caseload and the caseload where a VERA-2R has been used and assessed. For whatever reason, professionals are not able to use the VERA-2R regularly. The fact that professionals are not able to use the VERA-2R regularly might interfere with the quality of their risk reports. Specifically, once professionals are certified to use a certain risk assessment tool, they need regular practical work experience to develop their expertise and knowledge on how to write a risk assessment report with risk scenarios and risk management (17, 18, 33). In addition, if professionals have relatively little work experience with risk assessment tools, this may explain why they report problems with the interpretation and weighting of indicators and risk domains. As various risk assessment tools are based on SPJ, the weighting of risk indicators is important to formulate a final risk assessment. The final professional judgement does not depend on the amount of risk indicators present (9, 13, 15, 34). If professionals have little experience with the SPJ method for violent extremism risk assessments, it might be difficult for professionals to feel confident in granting value to the coding based on their professional judgement. Research has shown that professionals gain more confidence with risk assessment tools if they are given the opportunity to practice it regularly (36).

Less than half of the professionals who used the VERA-2R did receive organizational support in the form of supervision and half of them received intervision. This lack of organizational support does not only apply to the VERA-2R. Research shows that it is not common for professionals to receive professional feedback after conducting risk assessments and advising on risk management strategies (37). It is important to have guidance and organisational support for the risk assessment of violent extremism, as this is a relatively complex topic (13). To illustrate, after having received the VERA-2R training, professionals do receive guidelines and writing formats for the violent extremism risk assessment. The use of these guidelines is important to be safeguarded through organizational supervision and feedback (38). Also, organisations should engage in intervision such as updating the professional’s knowledge on the topic of violent extremism, practical guidance in the form of case discussions and possibility of consensus meetings.

In the Netherlands, presumed privacy issues seriously interfere with and complicate the transfer of risk information between organisations. Professionals of the Dutch Probation Board report to experience difficulties in the transfer of information, because they report that the information they do have is mostly outdated. Two out of the three Dutch terrorist prison wings have psychologists updating VERA-2R assessments during detention. Because these psychologists are licensed mental health professionals, it is assumed that the VERA-2R information should not be shared. This information is subsequently not necessarily transferred to the Dutch Probation Board when terrorist detainees qualify for (conditional) release. Although verbal transfer of risk information does take place between prison and probation, during an individual’s detention, this information does not find its ground in written reports. When risk information is verbally shared and does not find its ground in written reports, it will not be traceable and usable in a later stadium. Thus, other involved parties, including the suspect or detainee himself, will not be able to retrieve this information.

Moreover, during supervision and monitoring, the Dutch Probation Board and the probation office of the Swedish Prison and Probation Board do not (yet) conduct VERA-2R re-assessments. They, however, pronounced their intention to do so. Also, the French speaking part of Belgium, did not implement the VERA-2R in their Probation Board. The VERA-2R guidelines, but this also applies to risk assessment in general, do explicitly state that re-assessments are needed to keep track of potential risk change and for making adjustments in appropriate risk management strategies, interventions and monitoring (9, 10). It is therefore an important issue to tackle.

Besides the relevance of evaluating the user experience of risk assessment tools by professionals in their organisational context, it is also important to know how professionals report the outcomes of their risk analysis. In general, reports that contain risk assessment outcomes serve as a communication tool to substantiate advice for third party organisations such as courts and other professionals (21, 33, 35, 39, 40). Therefore, studying the quality of these risk assessment reports is highly relevant, when evaluating the user value of the VERA-2R. This will be described in another article.

### Limitations

This study also has several limitations. First of all, there was no high response rate on the survey for VERA-2R trained professionals. It is not known whether or not the trained professionals that not responded had the opportunity to work with the VERA-2R. However, we may expect that specifically the professionals who are interested in the VERA-2R have responded. Also, participants did report about the availability of supervision. In the interviews it was explained that supervision was available in some contexts, for example the NIFP and the Belgian Federal Public Service (FOD) supervise each extremist report. However, participants did not indicate this as supervision. They might have thought that supervision only involved the presence of a supervisor during the assessment or scoring of the VERA-2R.

### Recommendations

In sum, this study shows that using the VERA-2R comes with a variety of challenges, both on the professional and organisational level. VERA-2R trained professionals were given few opportunities to use the instrument and when they did, they were not always offered regular supervision, intervision and booster training. Also, organisational issues in collaboration between judicial partner organisations and the lack of risk transfer information to professionals came to light.

Implications for research involve the need for future practice related research into the needs for both violent extremism risk assessors and recipients of risk assessment reports to establish interorganizational and interprofessional good practice standards. As previously described, it is of particular importance that (violent extremism) risk assessment is sufficiently described and substantiated in reports, as these reports serve as a risk communication tool for third party organisations (18, 21, 27, 33, 39-41). We will discuss this in a future article in relation to intervention possibilities, ideology and psychopathology (42).

‘Real life’ validation research into the use of the VERA-2R is necessary for different judicial settings. This is also relevant for other risk assessment tools (18, 21, 24). Is the tool appropriate and usable for the judicial situation and context? We conducted research into the validity of the VERA-2R for the pre- en post-trial situation, and will present this in a future article.

We think that the subject of risk transfer should receive more attention both scientifically and in practice. We consider the following practical remarks and recommendations to be relevant for the professional and organisational use of the VERA-2R:

1. Develop booster training sessions for professionals in relation to their judicial context. This allows VERA-2R trained professionals to develop and maintain their expertise.
2. Develop more organizational support, in the form of intervision, supervision and professional feedback. This especially applies for countries or judicial contexts in which the extremist or terrorist caseload is low.
3. Develop clear guidelines within and between organizations on whether and how to reassess with the VERA-2R for changes in an individual’s legal or legal situation. For example, transfer to another prison unit, parole or conditional release from prison, supervision by the probation service. This research shows that reassessments are rarely or not at all applied and are often not available to judicial partners.
4. Improve the risk transfer between judicial organisations and judicial professionals. This is necessary for adequate risk management. This study has shown that risk transfers are often lacking. A good example is the risk transfer information for conditionally released terrorists from the Flemish prison with VERA-2R reassessments by the Flemish Probation Board.
5. Professionals who use VERA-2R assessments, for example, prosecutors and judges, may benefit from a short training or webinar on violent extremism and terrorism and violent extremism risk assessment and risk management.
6. Judicial organizations and professionals in different judicial contexts can benefit from clear writing guidelines for VERA-2R risk reports. This can be developed on the basis of general writing guidelines and VERA-2R manuals that come with the training.

## Data availability statement

The original contributions presented in the study are included in the article/Supplementary material, further inquiries can be directed to the corresponding author.

## Ethics statement

Ethical review and approval was not required for the study on human participants in accordance with the local legislation and institutional requirements. Written informed consent from the participants was not required to participate in this study in accordance with the national legislation and the institutional requirements.

## Author contributions

ND developed the concept and design of the study. CO carried out the research and wrote the first draft of the manuscript under supervision of ND. All authors contributed to the article and approved the submitted version.

## Funding

The research was funded for 2 years by the EU (JUST-JCOO-AG-2020, project number 101007383).

## Conflict of interest

The authors declare that the research was conducted in the absence of any commercial or financial relationships that could be construed as a potential conflict of interest.

## Publisher’s note

All claims expressed in this article are solely those of the authors and do not necessarily represent those of their affiliated organizations, or those of the publisher, the editors and the reviewers. Any product that may be evaluated in this article, or claim that may be made by its manufacturer, is not guaranteed or endorsed by the publisher.

## Footnotes

^1^ https://www.vera-2r.nl/

^2^See footnote 1.
