# Supplementary material for: Using the VERA-2R, professional and organisational aspects
Source: Front Psychiatry. 2023 Jul 20;14:1165279. doi: 10.3389/fpsyt.2023.1165279 (PMC10400441; doi:10.3389/fpsyt.2023.1165279)
Supplement: Supplementary file 1 [file Data_Sheet_1.docx]

**Appendix Questionnaire Use VERA-2R**

The Violent Extremist Risk Assessment tool (VERA-2R) offers evidence-based professionalism with indicators based on empirical and expert knowledge in radicalization, violent extremism and terrorism. The VERA-2R tool can be used to establish the risk status and risk management for terrorist offenders in prison, discharge from detention, and when they return into society.

This digital questionnaire will take about 10 minutes to fill out. It will include multiple choice and a few open questions about the VERA-2R’s usability, satisfaction and support of structured professional judgment. We would like to ask for your feedback and opinion on the use of the VERA-2R within your field of work. Data will be administered anonymously. After data collection and analysis, the results will be communicated in a confidential report.

1. Do you give consent for participation in this research?
   1. Yes
   2. No
2. **GENERAL QUESTIONS**
3. When did you follow the VERA-2R training?

O 2015

O 2016

O 2017

O 2018

O 2019

O 2020

O 2021

1. In which country do you work?

O Sweden

O Belgium – French part

O Belgium – Flemish part

O The Netherlands

O Germany

O Denmark

O France

O Finland

O UK

O Austria

1. What is your field of work?

O Intelligence

O Law

O Police

O Providing pre-trial advice

O Providing post-trial advice

O Prison differentiation Probation

O Research

1. What is your field of expertise?

O Intelligence expert

O Police officer

O Psychologist

O Psychiatrist

O Social Worker

O Prison official

O Law

O Research

O Other:

1. **RISK ASSESSMENT TOOLS AND SPJ**
2. How long do you work with risk assessment tools in general?

O I don’t do evaluations with risk assessment tools

O Less than 1 year

O 1-3 years

O 4-6 years

O 6-10 years

O 10 years or more

1. Do you work with risk assessment tools that use the SPJ method?
   1. Yes
   2. No

When indicated yes:

How often do you use these SPJ risk assessment tools?

O Daily

O Weekly

O Monthly

O Yearly

O Less than once a year

1. **VERA-2R ASSESSMENT**
2. How often did you use the VERA-2R within the last 2 years?

O Never

O Once

O 2-4 times

O 5-10 times

O 10-15 times

O 15 times or more

1. If you used the VERA-2R once or never, what is the reason for that?
2. I know how to use the VERA-2R

O Yes

O No

1. The VERA-2R SPJ method enables a good risk assessment and risk management of an individual

O Strongly agree

O Agree

O Neutral

O Disagree

O Strongly disagree

1. What aspects of the VERA-2R are helpful or not helpful to you?
2. **VERA-2R TRAINING**
3. The VERA-2R training was useful for me

O Strongly agree

O Agree

O Neutral

O Disagree

O Strongly disagree

1. After the VERA-2R training I knew how to use the tool in practice

O Strongly agree

O Agree

O Neutral

O Disagree

O Strongly disagree

1. After the VERA-2R training, did you receive supervision within your organization?

O Yes

O No

When indicated yes:

Specify the form and frequency of supervision:

1. Did or do you have VERA-2R intervision meetings (discussing a case with a peer group) possibilities within your organization?

O Yes, specify the form and frequency of the intervision:

O No

17A. Do you need VERA-2R supervision could be beneficial?

O Yes

O Probably

O No

17B. Do you think VERA-2R intervision could be beneficial?

O Yes

O Probably

O No

1. Continuous learning is needed for using the VERA-2R

O Strongly agree

O Agree

O Neutral

O Disagree

O Strongly disagree

1. For which aspects do you think continuous learning is necessary?

O Literature

O Practice

O SPJ-method

O Psychopathology

O Risk scenario’s

O Risk reporting

O Risk communication

O Risk management

O Right-wing extremism

O Left-wing extremism

O Lone actors

O Other, specify

O None

1. **VERA-2R REPORTING**
2. Do you use the NIFP based VERA-2R writing format?

O Yes

O Probably

O No

1. Does you use an own VERA-2R writing format?

O Yes, specify:

O No

1. When indicated yes: Does your own writing format contain risk management aspects in addition to the risk analysis?

O Yes

O No

1. **VERA-2R PERSONAL USER EXPERIENCE**
2. The VERA-2R is my standard tool for violent extremism risk assessment and risk management of a subject

O Yes

O No, specify other tools you use:

1. The VERA-2R helps to structure the necessary information on violent extremism risk assessment and risk management of a subject

O Strongly agree

O Agree

O Neutral

O Disagree

O Strongly disagree

1. How much time do you spend on a VERA-2R assessment?

O 2 hours or less

O 3 hours

O 4 hours

O 5 hours

O 6 hours or more

1. The VERA-2R can be made more useful for me as a professional

O Yes, clarify how:

O No

1. I have enough time and opportunity to use the VERA-2R in my working situation

O Strongly agree

O Agree

O Neutral

O Disagree

O Strongly disagree

1. If you have not enough time and/or the opportunity to use the VERA-2R in your working situation, how can this be changed?
2. **VERA-2R WITHIN YOUR ORGANIZATION**
3. The VERA-2R is considered useful within my organization

O Strongly agree

O Agree

O Neutral

O Disagree

O Strongly disagree

1. The management of my organization stimulates the use of the VERA-2R

O Strongly agree

O Agree

O Neutral

O Disagree

O Strongly disagree

1. We are able to have VERA-2R consensus meetings with a collague on a case within my organization

O Strongly agree

O Agree

O Neutral

O Disagree

O Strongly disagree

1. Using the VERA-2R enhances the risk communication about subjects with professionals in my organisation

O Strongly agree

O Agree

O Neutral

O Disagree

O Strongly disagree

1. What can be changed to enhance the risk communication regarding the VERA-2R within your organization?
2. **RISK COMMUNICATION**
3. Do you communicate risks or risk management outside your organization, using the VERA-2R?

O Yes

O No

When indicated yes:

1. Using the VERA-2R enhances the risk communication about subjects with professionals outside my organization

O Strongly agree

O Agree

O Neutral

O Disagree

O Strongly disagree

1. What could be changed to enhance risk communications with professionals outside my organization, regarding the VERA-2R?
2. Do you have any additional comments on the use of the VERA-2R?
